# Intestinal release of biofilm-like microcolonies encased in calcium-pectinate beads increases probiotic properties of *Lacticaseibacillus paracasei*

**DOI:** 10.1038/s41522-020-00159-3

**Published:** 2020-10-28

**Authors:** Arnaud Heumann, Ali Assifaoui, David Da Silva Barreira, Charles Thomas, Romain Briandet, Julie Laurent, Laurent Beney, Pierre Lapaquette, Jean Guzzo, Aurélie Rieu

**Affiliations:** 1 Université de Bourgogne Franche-Comté (UBFC), AgroSup Dijon, UMR PAM A 02.102, F-21000 Dijon, France; 2 Université de Bourgogne Franche-Comté (UBFC), LNC UMR 1231, F-21000 Dijon, France; INSERM, LNC UMR 1231, F-21000 Dijon, France; 3 Université de Bourgogne Franche-Comté (UBFC), LipSTIC LabEx, F-21000 Dijon, France; 4 Université Paris-Saclay, INRAE, AgroParisTech, Micalis Institute, 78350 Jouy-en-Josas, France

**Keywords:** Biofilms, Cellular microbiology

## Abstract

In this study, we show that calcium pectinate beads (CPB) allow the formation of 20 µm spherical microcolonies of the probiotic bacteria *Lacticaseibacillus paracasei* (formerly designated as *Lactobacillus paracasei*) ATCC334 with a high cell density, reaching more than 10 log (CFU/g). The bacteria within these microcolonies are well structured and adhere to a three-dimensional network made of calcium-pectinate through the synthesis of extracellular polymeric substances (EPS) and thus display a biofilm-like phenotype, an attractive property for their use as probiotics. During bacterial development in the CPB, a coalescence phenomenon arises between neighboring microcolonies accompanied by their peripheral spatialization within the bead. Moreover, the cells of *L. paracasei* ATCC334 encased in these pectinate beads exhibit increased resistance to acidic stress (pH 1.5), osmotic stress (4.5 M NaCl), the freeze-drying process and combined stresses, simulating the harsh conditions encountered in the gastrointestinal (GI) tract. In vivo, the oral administration of CPB-formulated *L. paracasei* ATCC334 in mice demonstrated that biofilm-like microcolonies are successfully released from the CPB matrix in the colonic environment. In addition, these CPB-formulated probiotic bacteria display the ability to reduce the severity of a DSS-induced colitis mouse model, with a decrease in colonic mucosal injuries, less inflammation, and reduced weight loss compared to DSS control mice. To conclude, this work paves the way for a new form of probiotic administration in the form of biofilm-like microcolonies with enhanced functionalities.

## Introduction

The human gut harbors a complex ecosystem composed of 10^13–14^ microorganisms. Non-pathogenic commensal microbiota is necessary for normal gastrointestinal (GI) physiology. Lactobacilli are common residents of the GI tract microbiota of mammals and some strains are considered as potential probiotics. The World Health Organization (WHO) has defined probiotics as ‘live microorganisms which, when administered in adequate amounts confer a health benefit on the host’^1^. Several probiotics have been demonstrated to enhance GI health, in part by stimulating host immunity and inhibiting pathogen adherence to mucus and intestinal epithelial cells^2,3^. Probiotic bacteria, which are administered orally, have to cope with the adverse conditions encountered in the GI tract and their release must be controlled to ensure maximum effectiveness^4,5^. Due to their non-toxicity, biocompatibility, and low cost, polysaccharides such as alginate and pectin are used to encapsulate probiotics^6^. Pectin is a polysaccharide rich in D-galacturonic acid and contains significant amounts of neutral sugars such as D-xylose, D-glucose, L-rhamnose, L-arabinose, and D-galactose. It was shown that calcium pectinate beads (CPB) are resistant to gastric acid and are specifically degraded by bacterial enzymes in the colon, allowing their use as vectors for colon-targeted molecule delivery^7,8^. Calcium-pectinate beads which are formed when a drop of pectin solution is in contact with calcium ions (external gelation), have the ability to entrap and immobilize bacteria in their 3D network, improving their resistance to external stress and therefore their viability^4,9,10^. This gelation protocol induces an anisotropic gel whose polymer concentration and rigidity decrease from the periphery to the core of the bead^11^. These phenomena have already been explained by the presence of a considerable gradient of the mesh size, which increases drastically from the periphery (~10 nm) to the core of the bead (~10 µm)^12,13^.

Another way to protect bacteria from environmental stress is biofilm culture. Biofilms are communities of microorganisms associated with a surface and commonly embedded in extracellular polymeric substances (EPS)^14^. In such communities, microorganisms generally demonstrate increased tolerance to harsh environments^15,16^. It is recognized that the biofilm matrix plays a key role in the resistance of these communities by, for instance, limiting the diffusion of toxic molecules^17,18^. In addition, the development of a complex three-dimensional (3D) biofilm architecture results in the formation of nutrient, gas and signaling molecule gradients^19^. This leads to differential gene expression and specific physiological activities throughout the biofilm in response to local micro-environmental conditions^17^. Therefore, the emergence of phenotypic variants in biofilm subpopulations contributes to the expression of novel community functions such as tolerance to environmental stress^18^. These phenotypic variants can also express new molecules or a larger number of molecules than their planktonic (i.e. free-floating cells) counterparts; i.e., polysaccharides, surface proteins, surface appendages, secreted proteins, and peptides, many of which are capable of modulating the activities of host cells^20,21^. Our group described the ability of several strains of *Lactobacillaceae* to form biofilm that exacerbates their beneficial effects to host cells^22,23^. Indeed, *Lactobacillaceae* biofilms are resistant to GI environment-related conditions and produce large amounts of extracellular factors with immunomodulatory properties^22^. The supernatant of *Lactobacillaceae* grown as biofilm is more potent for damping inflammation induced by LPS challenge in human macrophages, compared to the supernatant from the same bacteria grown planktonically^23^. *Lacticaseibacillus paracasei* ATCC 334, the strain chosen for this study, has been shown to have its immunodulatory activities enhanced when grown as a biofilm^23^.

In this study, we combined (i) the ability of *L. paracasei* to form a biofilm on a surface, and (ii) the properties of calcium-pectinate beads to encapsulate and control the release of encased bacteria. We hypothesized that the 3D network formed by calcium-pectinate beads plays the role of a surface on which bacteria can adhere and grow in biofilm form. We first immobilized *L. paracasei* ATCC334 in calcium-pectinate beads (CPB), and then these beads were introduced in specific cultural medium allowing bacterial growth. After incubation, the protective effects of both the pectinate matrix and the biofilm-like microcolonies inside these beads were evaluated in stress resistance models (in vitro) and in an acute DSS-induced colitis model in mice (in vivo). Traits of the CPB-formulated biofilm of *L. paracasei* ATCC334 were compared to the traits of planktonic and surface-associated biofilm cultures.

## Results

### Development of biofilm-like microcolonies encapsulated in calcium-pectinate beads

**Fig. 1:**
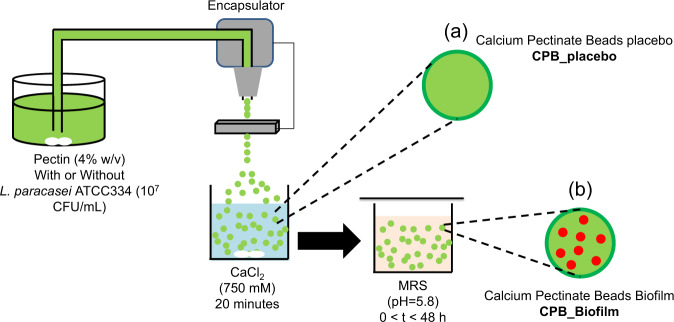
Schematic illustrations of the experimental protocol for the production of calcium-pectinate beads (CPB). The formation of the beads is based on the ionotropic gelation due to the interaction between the pectin solution and calcium ions. Buchi Encapsulator (B-390) was used to produce beads with a small size (frequency vibration = 700 Hz, liquid flow rate = 12 mL/min, the diameter of the used nozzle = 300 µm). **a** The beads CPB_placebo are prepared without the presence of *L. paracasei* ATCC334 and after a curing time of 20 min. **b** The beads containing *L. paracasei* ATCC334 are incubated in MRS (pH = 5.8) for different times (from 0 to 48 h) to allow the formation of biofilm-like microcolonies inside the beads (CPB_Biofilm).

**Fig. 2:**
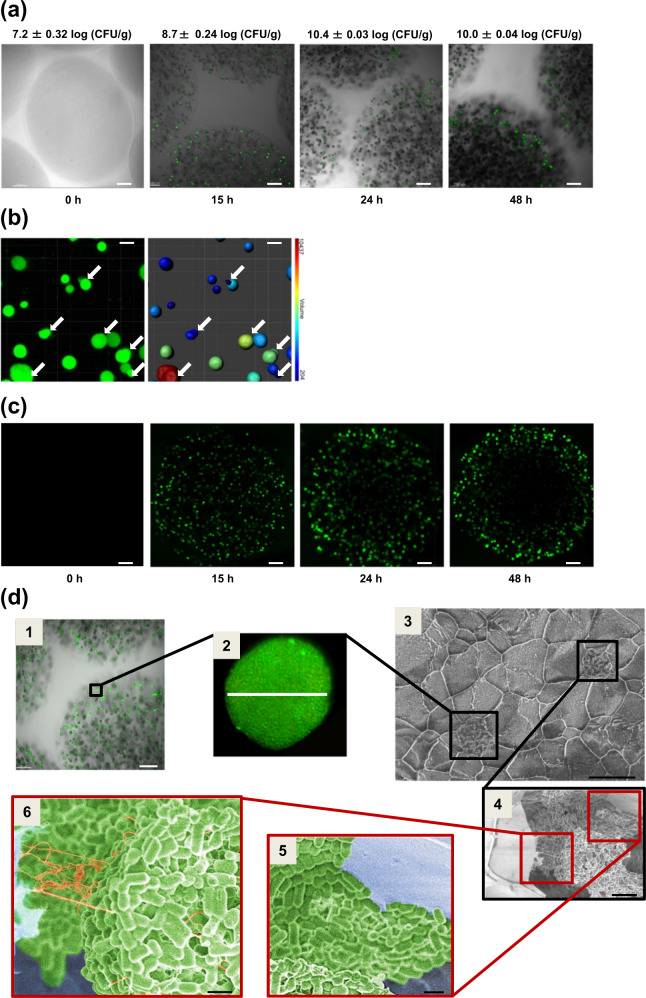
Development of *L. paracasei* ATCC334 biofilm-like microcolonies encapsulated in pectin beads. **a**–**c** Images of CPB_Biofilm obtained by laser scanning confocal microscopy (CLSM) at different incubation times in MRS medium. **a** CPB is contrasted in the transmitted light channel in gray. Bacterial cells were labeled with Syto 9 green-fluorescent nucleic acid stain (scale bars, 100 μm). **b** In order to visualize size diversity and microcolony coalescence, fluorescence images obtained at 15 h were transformed into isosurface representations with a coded color associated with microcolony volume (scale bars, 20 μm). **c** The evolution of the microcolonies’ spatial distribution in a CPB during 48 h of incubation in MRS. The initial homogeneous distribution of fluorescent colonies at 15 h gave way to their peripheral distribution and the emergence of a central hollow void in the beads after 24 and 48 h of incubation (scale bars, 100 μm). **d** Microcolony organization inside the pectin beads of CPB_Biofilm after an incubation time of 24 hours in MRS medium. Images obtained by CLSM (1 and 2) and cryo-SEM (3, 4, 5, and 6). Black boxes indicate biofilm-like microcolonies (1, 2, 3, and 4) and red boxes highlight the adhesion of bacteria to the matrix of pectin beads (5) and the presence of extracellular polymeric substances in the microcolonies (6). Images 5 and 6 have been colorized. Scale bars, (1) 100 μm, (2) 20 μm, (3) 20 μm, (4) 5 μm, (5) 1 μm and (6) 1 μm.

We investigated the ability of *L. paracasei* ATCC334 to develop biofilm inside calcium-pectinate beads (CPB). The schematic of the technique to form CPB is shown in Fig. 1. Therefore, we immobilized bacteria (10^7^ CFU/g) in CPB before incubation for up to 48 h in MRS culture medium, without shaking. As the incubation time increased, the number of bacteria enumerated increased to reach a maximal value exceeding 10.4 log (CFU/g) after 24 h (Fig. 2a). The CLSM images show a distribution of small green rounded-shape volumes with a typical diameter of 20 µm inside the CPB bead visualized on the gray transmitted light signal. These green spots correspond to microcolonies of *L. paracasei* ATCC334 labeled beforehand with the fluorescent cell-permeant Syto 9 (Fig. 2a).

Further image analysis allowed us to determine the number (*N*) and the surface area (*S*) of these microcolonies (Table 1) as a function of the incubation time. The results showed that the number of microcolonies decreased drastically over time (from *N* = 215 at 15 h to *N* = 92 at 48 h) at the expense of their surface area. Indeed, the surface area increased more than 10 times between 15 and 48 h (Table 1). The decrease of the number of microcolonies in time was likely due to their merging when they came into contact with each other, thus increasing the surface area measured. This hypothesis was supported by CLSM images at 15 h which showed coalescence phenomena between neighboring microcolonies (Fig. 2b). The same observations of merging between neighboring microcolonies were made at 24 and 48 h (Supplementary Fig. 1). This coalescence increased microcolony sphericity over time, as indicated by an increase in the sphericity value from 0.47 at 15 h to 0.77 at 48 h. Moreover, the total surface of biofilm-like culture in one bead, which can be considered as the product of the surface area of microcolonies (*S*) and their number (*N*), increased with incubation time (Table 1). This increase indicates that, in addition to the microcolony merging phenomenon, bacteria continued growing with incubation time. In addition, CLSM revealed the emergence of a peripheral spatialization of the microcolonies within the CPB during incubation (Fig. 2c). Indeed, a random distribution of microcolonies throughout the CPB was observed at an incubation time of 15 h while at 24 and 48 h only a few microcolonies were detected in the center of the bead. Although the reorganization of the distribution of microcolonies inside the bead changed between 24 and 48 h, we did not notice significant changes in enumerated bacteria which reached a maximum value exceeding 10 log (CFU/g).

**Table 1:** Quantitative parameters extracted from mosaic images obtained at different times using the Imaris software.

Parameters	15 h	24 h	48 h
Number of microcolonies (*N*)/bead^a^	215	208	92
Surface area of microcolonies (*S*) (µm^2^)	537 ± 31	1119 ± 44	6941 ± 51
Sphericity of microcolonies	0.47 ± 0.06	0.45 ± 0.08	0.77 ± 0.05
Total of biofilm-like/bead (µm^2^)^b^	1.15 × 10^5^	2.33 × 10^5^	6.39 × 10^5^

^a^Obtained after analysis of 10 beads.

^b^Calculated as the product of the number (*N*) per surface (*S*) of microcolonies.

Moreover, cryo-SEM images confirmed the CLSM observations showing the incubation of the bacteria in 20 µm microcolonies distributed in the CPB after 24 h (Fig. 2d 1, 2, and 3). In addition, cryo-SEM showed that bacteria adhered closely to the wall of the calcium-pectinate network (Fig. 2d 4 and 5). Furthermore, cryo-SEM images indicated the existence of fibers connecting bacteria to each other but also connecting microcolonies to the surface of the 3D calcium-pectinate network (Fig. 2d 4 and 6). Based on previous reports in the literature^24^, such fibers might correspond to the EPS synthesized by the bacteria during their growth in a biofilm-like mode of life. For the following experiments, we worked on the beads which were incubated for 24 h, presenting a maximal value of 10.4 log (CFU/g).

### Stress resistance of *L. paracasei* ATCC334 biofilm-like microcolonies

**Fig. 3:**
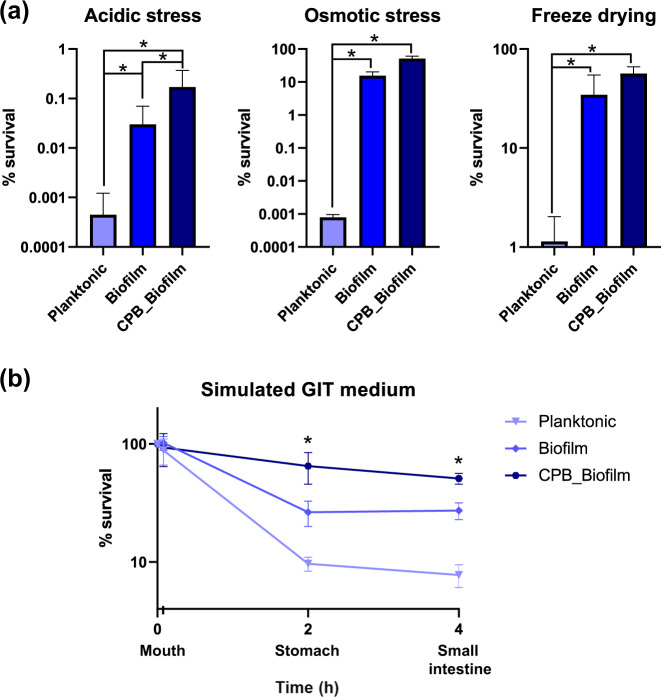
Resistance of *L. paracasei* ATCC334 to several stresses. Survival of *L. paracasei* ATCC334 in planktonic form, biofilm form and encapsulated as biofilm-like cultures in calcium-pectinate beads (CPB_Biofilm) after (**a**) acidic stress, osmotic stress, freeze-drying and (**b**) incubation in simulated GIT medium (mouth, stomach, and small intestine). Results are expressed as the percentage of viable bacteria measured after stress exposure. The initial amount of bacteria subjected to stress is 100%. Each value is the mean of at least three independent experiments. Asterisks indicate statistically significant differences (*P < 0.05), (Kruskal–Wallis test).

We evaluated the resistance, in terms of bacterial survival, of CPB-formulated biofilm in response to stresses encountered in the GI tract (acidic stress), in food (osmotic stress), and in industrial processes (freeze-drying) (Fig. 3a). In the three cases, stress drastically reduced the viability of planktonic-grown bacteria while in comparison the survival of bacteria under biofilm and CPB_Biofilm conditions remained significantly higher. We also tested the resistance of these three culture conditions in successive simulated GI fluids, reproducing the main features of the physico-chemical environments of the mouth, stomach, and small intestine (Fig. 3b). As expected, increased bacterial mortality was observed after the acidic stress occurring in the stomach-mimicking medium. However, CPB-formulated biofilm-like microcolonies coped better with this stress, with almost no decrease in their survival compared to planktonic and biofilm-grown *L. paracasei* ATCC334 cells. The cells inside the CPB_Biofilm survived stress that mimicked the conditions encountered in the small intestine (low pH, high concentration of bile salts and enzymes) and were able to reach the duodenal compartment in sufficient amounts, with a limited population loss. Altogether, these results demonstrated that CPB_Biofilm growth allows better resistance of *Lactobacillaceae* to the various stresses encountered in the GI tract.

### Survival and implantation of *L. paracasei* ATCC334 biofilm-like microcolonies in DSS-treated mice

**Fig. 4:**
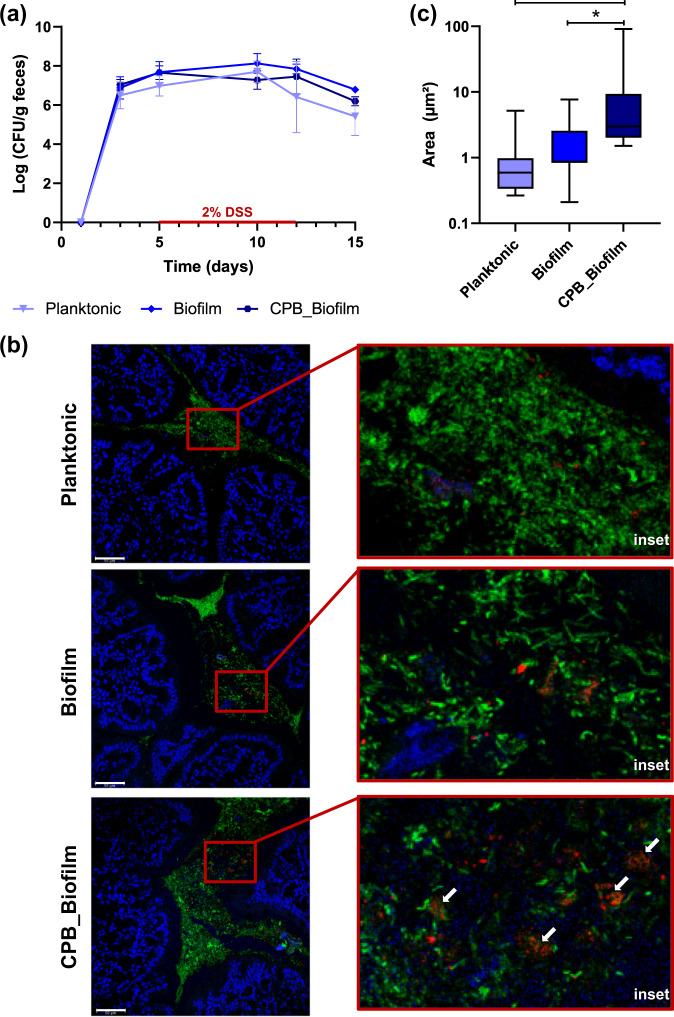
Survival and implantation of *L. paracasei* ATCC334 in the colon of DSS-treated mice. **a** *L. paracasei* cells in mouse feces were enumerated every second day of the study on M-RTLV agar. The results presented are representative of 8 mice from each group (lower limit of detection, 2.5 log (CFU/g) feces). **b** In situ hybridization with fluorescence-labeled oligonucleotide probes. Colon sections of mice were hybridized with a *Lactobacillaceae*-specific oligonucleotide probe (red) and a bacterium-specific oligonucleotide probe (green). *L. paracasei* ATCC334 cells appear red while all other bacteria appear green. Host nuclei were labeled with DAPI (blue) (scale bars, 50 μm). **c** Size distribution of *L. paracasei* foci observed on FISH images. The number of images analyzed was five for each condition. Asterisks indicate statistically significant differences (*P < 0.05), (Kruskal–Wallis test).

Next, we investigated the survival of biofilm-like microcolonies encased in CPB that were administered orally to mice. *L. paracasei* cells were enumerated in feces using selective M-RTLV agar (Fig. 4a). Prior to the administration of *L. paracasei* ATCC334, the concentration of *L. paracasei* in mouse feces were below the detection limit (<2.5 log (CFU/g feces)). In mice receiving the probiotic, the enumerations of viable *L. paracasei* ATCC334 cells in the feces confirmed that the bacteria survived throughout the GI tract prior to and during DSS administration. No significant difference in CFU in the feces was noticed between the three growth conditions of *L. paracasei* ATCC334 after their administration in mice. We then used FISH to investigate the implantation of *L. paracasei* ATCC334 in the colon of force-fed mice (Fig. 4b). The colonic microbiota that hybridized with the universal bacterial probe (green) was present in a large amount in the intestinal lumen above the mucus layer produced by the goblet cells. In control mice, the detection of *Lactobacillaceae* with a dedicated probe (red) revealed a low representation of endogenous lactobacilli within the total microbiota (Supplementary Fig. 2). In the colon of mice that received planktonic or biofilm-grown *L. paracasei* ATCC334, bacteria that hybridized with the *Lactobacillaceae* probe were mainly dispersed (Fig. 4b). By contrast, in mice force-fed with *L. paracasei* ATCC334 in the CPB_Biofilm, bacteria appeared in clusters. Assessment of the size of bacterial foci observed on FISH images revealed a significant increase of foci size measured for the CPB_Biofilm-treated mice, compared to planktonic and biofilm-treated mice (Fig. 4c). Some clusters observed in the colon of CPB_Biofilm-treated mice were about 20 µm in diameter, similar to the biofilm-like microcolonies observed inside the pectin beads (Fig. 2a). These results indicate that *L. paracasei* ATCC334 cells are released as biofilm-like structures in the mouse colon by CPB_Biofilm.

### Protection against DSS-induced colitis in mice by *L. paracasei* ATCC334 biofilm-like microcolonies

**Fig. 5:**
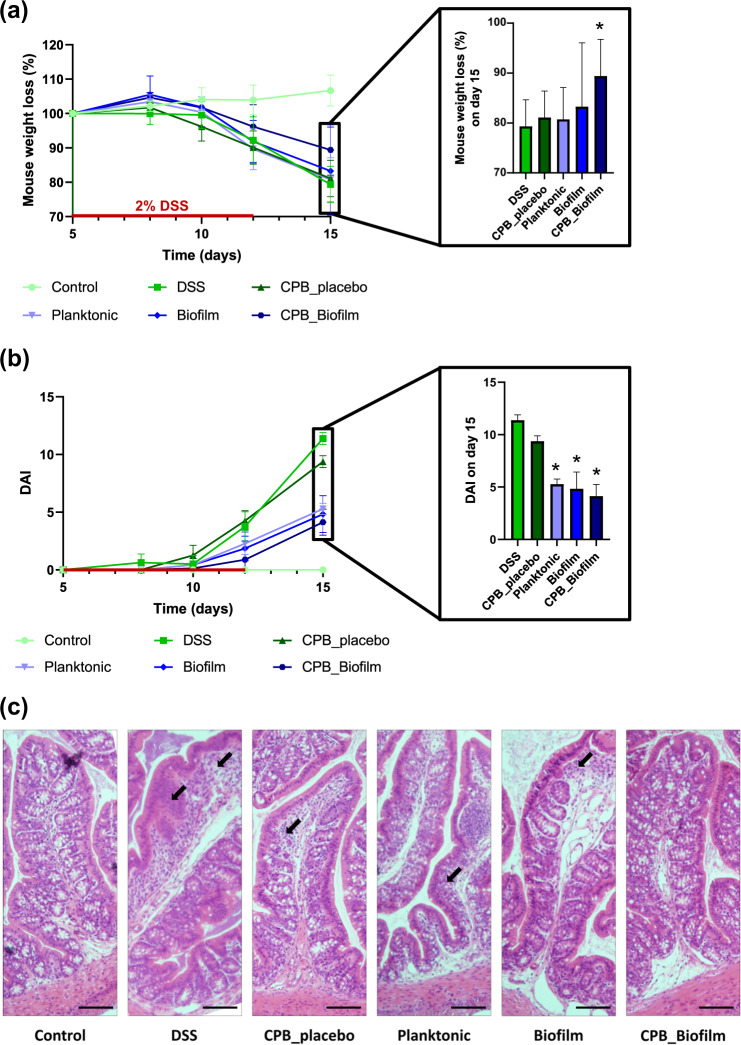
Evaluation of treatment efficacy by biofilm-like *L. paracasei* ATCC334 encapsulated in pectin beads in DSS-induced colitis mice. The results presented are representative of 8 mice from each group. Asterisks indicate statistically significant differences from the DSS-induced colitic group (*P < 0.05), (Kruskal–Wallis test). **a** Changes in mouse body weight during and after DSS administration. The percent change in body weight was calculated by using the weight on day 5 (the day of DSS initiation) as the reference. **b** Clinical assessment of DSS-induced colitis. Disease Activity Index (DAI) was scored from each mouse for weight loss, stool consistency, and bleeding. **c** Histologi**c**al sections of H&E-stained colon of representative mice from each group (tissue damage is shown by arrows) (scale bars, 30 μm).

To determine if CPB-formulated biofilm of *L. paracasei* ATCC334 exerted anti-inflammatory properties in vivo, we conducted animal experiments using a well-characterized mouse model of intestinal inflammation (DSS-induced colitis)^25^. As expected, DSS-colitic mice (DSS) presented a considerable weight loss (21%). By contrast, weight loss was significantly less pronounced (11%) in mice treated with the CPB-formulated biofilm of *L. paracasei* ATCC334 (Fig. 5a). Of note, the other formulations of *L. paracasei* (Planktonic and Biofilm) were not able to limit the DSS-induced weight loss, suggesting a protective effect of the CPB_Biofilm formulation. Notably, *L. paracasei* ATCC334-treated mice showed a significantly lower disease activity index (DAI) compared to DSS-treated mice, indicating a better outcome regarding the parameters of stool consistency and blood in the stool (Fig. 5b). Besides weight loss and the DAI score, mice treated with *L. paracasei* ATCC334 in CPB_Biofilm presented fewer histological signs of tissue damage (Fig. 5c). Indeed, DSS-treated mice (positive control) developed severe tissue damage in their colons, characterized by the loss of crypts, the erosion of the epithelial monolayer and infiltration of inflammatory cells. By contrast, the colons of mice receiving the CPB-formulated *L. paracasei* ATCC334 (CPB_Biofilm) showed reduced signs of tissue damage with preserved crypt structures and decreased immune cell infiltration compared to DSS-treated mice. Mice receiving planktonic cells (Planktonic) and surface-associated biofilm cells (Biofilm) also demonstrated less histological damage in comparison with DSS-treated mice; however, there were still large infiltrations of inflammatory cells and a loss of goblet cells in their colons. Altogether, our observations evidenced that *L. paracasei* ATCC334 biofilm-like microcolonies provide an increased protective effect on the GI tract during DSS-induced colitis compared to planktonic and surface-associated biofilm cultures.

### Reduction of inflammation in mice by *L. paracasei* ATCC334 biofilm-like microcolonies

**Fig. 6:**
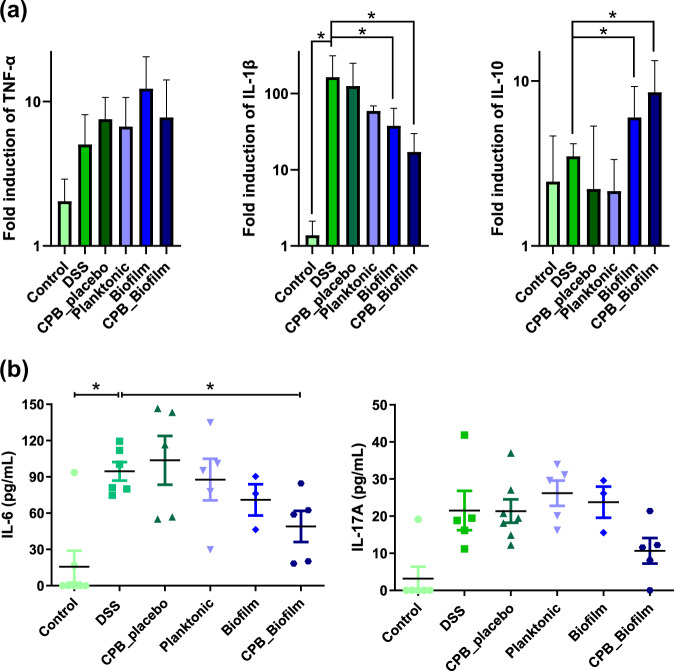
Modulation of mice inflammation by biofilm-like *L. paracasei* ATCC334 encapsulated in pectin beads. Asterisks indicate statistically significant differences from the DSS-induced colitic group (*P < 0.05), (Kruskal–Wallis test). **a** The expression of TNF-α, IL-1β and IL-10 was analyzed by qRT-PCR in mice colons. The results were determined by the comparative critical threshold (CT) method. The GAPDH gene was used as the internal standard, and the expression of genes in the non colitic control group was used as the calibrator. **b** Plasma levels of Il-6 and Il-17A were measured simultaneously using a multiplex assay from plasma of mice.

In order to confirm the protective effects of CPB-formulated *L. paracasei* during DSS-induced colitis, we next evaluated the immune response by measuring the expression of various cytokines. First, we used RT-qPCR to measure the mRNA levels of genes encoding pro- and anti-inflammatory cytokines in the mouse colon at the end of the experiment (Fig. 6a). The levels of transcripts of the pro-inflammatory cytokines IL-1β were significantly higher in DSS-treated mice compared to untreated mice. For mice receiving biofilm-like microcolonies encased in CPB (CPB_Biofilm) or biofilm-grown *L. paracasei* ATCC334 (Biofilm) before and during DSS exposure, the levels of IL-1β transcripts were significantly lower than in mice treated with DSS alone or treated with planktonic-grown *L. paracasei* ATCC334 (Planktonic). No significant difference was observed for the levels of TNF-α transcripts between any of the DSS-treated conditions. Interestingly, the mRNA levels of the gene encoding the anti-inflammatory cytokine IL-10 were higher in mice treated with the biofilm-like microcolonies encased in CPB or biofilm-grown *L. paracasei* ATCC334 compared to the other conditions. We then used a multiplex assay to simultaneously measure 6 relevant cytokines at the protein level (IL-1β, IL-6, IL-10, IL-17, IFN-γ, TNF-α) from the plasma of mice (Fig. 6b). DSS-treated mice displayed a significant increase in plasma levels of the pro-inflammatory cytokine IL-6 compared to control mice. Interestingly, a significant decrease in IL-6 level was observed in the CPB_Biofilm-treated group compared to DSS-treated mice (DSS). Although non-significant, the levels of IL-17, a pro-inflammatory cytokine, in the CPB_Biofilm-treated group also tended to be reduced in comparison to the other DSS-treated groups. The amounts of the other four cytokines were below the detection limits. These results further confirm the anti-inflammatory properties of biofilm-like microcolonies encased in CPB in the DSS-induced colitis mouse model and could partially explain their ability to alleviate DSS-induced colonic damage (Fig. 5).

## Discussion

**Fig. 7:**
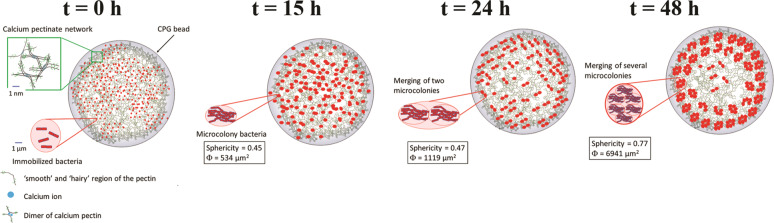
Bacterial growth model as biofilm-like microcolonies inside the CPB. At *t* = 0 h, bacteria were distributed randomly inside the pectinate bead and adhered to the walls formed by the 3D calcium-pectinate network. At *t* = 15 h, adherent bacteria were grown as microcolonies (biofilm-like) distributed randomly in the bead. At *t* = 24 h, a reorganization of microcolonies occurred with then merging of two microcolonies with each other associated with bacterial growth within the microcolonies. This led to a decrease in the number of microcolonies and an increase in their surface and their sphericity. At *t* = 48 h, the merging of several microcolonies continued to occur and the distribution of microcolonies became less random and more concentrated at the periphery of the bead.

In this article, we developed a colon-targeted delivery system of bacterial biofilm based on calcium-pectinate beads that encapsulate and control the release of *L. paracasei* biofilm. First, we immobilized *L. paracasei* ATCC334 (10^7^ CFU/g) in calcium-pectinate beads by ionotropic gelation. After this immobilization step (*t* = 0 h), calcium-pectinate beads containing bacteria were then incubated in MRS medium at 28 °C for 15, 24, and 48 h. At 15 h, the microcolonies were large enough to be observable by CLSM and were distributed randomly inside the bead. As the incubation time increased (*t* > 15 h), we observed that the microcolonies merged, leading to the increase of both their surface area and sphericity. In addition, the distribution of microcolonies became less random and more concentrated on the periphery of the bead with incubation time. The size of the microcolonies measured inside the beads by microscopic observations was homogeneous at each incubation time studied. Based on our results we propose the scenario presented in Fig. 7 for the biofilm-like bacteria growth inside the bead. In this model, bacteria were first distributed randomly inside the whole of the calcium-pectinate bead. During the first incubation times (0 < *t* < 15 h), bacteria were immobilized and then adhered to the walls formed by the 3D calcium-pectinate network before growing as microcolonies distributed randomly in the bead. In parallel, the bacteria synthesized EPS ensuring their adherence to each other as well as the wall. As the incubation time increased, the microcolonies reorganized, with a concentration of microcolonies (biofilm-like) at the periphery of the calcium-pectinate bead. This radial organization could have been due to the reduced diffusion of nutrients to the bacteria located at the core of the bead, possibly reducing the ability of bacteria to adhere and thus form biofilm-like microcolonies there. Studies of surface-adherent biofilms revealed a high level of heterogeneity, with complex chemical gradients and physiologically distinct subpopulations^18,26–28^. These studies lead us to suggest that this spatial organization could be related to the installation of radial nutritional gradients within the beads (i.e., some nutrients were consumed by bacteria on the periphery faster than they could be diffused throughout the bulk of the bead). As a matter of fact, previous studies described that the concentration of a substrate consumed decreased with depth in the biofilm and conversely, a metabolic product was more concentrated inside the biofilm^18^. Waste produced by *L. paracasei* ATCC334 during growth can have an inhibitory effect unless it is eliminated by diffusion. Otherwise, these waste products might accumulate at the center of the bead. This phenomenon could benefit bacteria by establishing a heterogeneous population prepared to endure rapid changes in its environment, including the environment of the GI tract.

Using an in vitro model, our study showed increased resistance of *L. paracasei* ATCC334 biofilm cultures to acidic stress (pH 1.5), osmotic stress (4.5 M NaCl) and the freeze-drying process in comparison to planktonic cultures. These results are in agreement with those of numerous studies demonstrating that biofilms are a mechanism of bacterial resistance, including resistance to the GI tract and industrial process stresses^22,29–33^. This phenomenon also occurs with lactobacilli biofilms^22^. The reason for this enhanced resistance can be attributed to several factors such as the restricted penetration of antimicrobial agents, reduced growth rates, the activation of the general stress response, the induction of a biofilm phenotype, and cell-to-cell communication (quorum sensing)^29–31,33^. In addition to the resistance properties conferred by biofilm growth, pectin provided increased resistance to the encased bacteria, especially against acidic stress. Indeed, the results showed an increase in the acidic stress resistance of bacteria in CPB_Biofilm compared to biofilm cells. This suggests a cumulative effect of biofilm and pectin beads to protect bacteria. Microcapsules of chitosan-coated alginate and microcapsules of carrageenan containing high-density biofilm-like *L. rhamnosus* GG probiotics have already been developed^34^. The encapsulated *L. rhamnosus* GG strain in biofilm form with a double coating was shown to be more resistant than the double-coated planktonic strain regarding exposure to stresses commonly encountered during industrial food processes (i.e., heating and freeze-drying)^34^. However, enhanced viability was not observed during refrigerated storage and exposure to simulated gastric juice^34^. Later, it was observed that alginate mixed with chitosan and locust gum provided the best resistance to stressful environments and also increased probiotic viability in a simulated gastric juice^35^. In our study, pectin provided biofilm cells with protection against acidity without the need for an additional layer. By providing protection against acid stress, pectin represents a polymer of choice for protecting probiotics from GI stress^10^. The physical barrier formed by pectin improves stress resistance during transit in the GI tract and during storage^4^. Similarly, pectin provided additional resistance to bacteria in biofilm form in the in vitro model of intestinal simulation. Indeed, the bacteria within the CPB_Biofilm exhibited additional survival capability in the three successive simulated GI fluids: mouth, stomach, and small intestine, suggesting a release of bacteria in the intestine with an increase in the number of viable cells released, ensuring better efficiency of health-promoting bacteria.

To evaluate the in vivo effects of encapsulation and biofilm phenotype on the viability of *L. paracasei* ATCC334, mice were force-fed with bacteria (planktonic, biofilm and CPB_Biofilm) and treated with DSS to induce colitis. Our results highlighted that DSS-treated mice supplemented with biofilm-like microcolonies encased in the pectin beads displayed a decrease in colonic mucosal injuries, lower inflammation and reduced weight loss compared to control mice and to a lesser extent to mice receiving *L. paracasei* without the CPB formulation. Numerous studies demonstrated a significant effect of probiotic efficacy using a DSS-induced colitis model in mice^4,36–41^. For instance, encapsulated *L. rhamnosus* GG exerted a reduced level of DSS-induced colonic injury compared to non-encapsulated *L. rhamnosus* GG^4^. In our study, no difference was observed in terms of the quantity of bacteria in feces between the three conditions (Planktonic, Biofilm, CPB_Biofilm), suggesting that the formulation used did not have an impact on the bacterial viability in the GIT of the mice. This last result may seem surprising since biofilm displayed a higher survival rate to in vitro GIT stress exposure. However, these discrepancies might be explained by the fact that simulated GIT media only reproduced physico-chemical parameters of the GIT, and did not mimic all the features of the mice GIT such as the resident gut microbiota and the mucus layer. In addition, the fact that the probiotics were daily administered (10^9^ CFU) might hide viability differences since new viable bacteria were daily introduced in the mice GIT. These results are in contrast to those of another study describing that the enhanced beneficial properties of the encapsulation system developed was directly linked to a higher survival rate of *L. rhamnosus* GG in the GIT^4^. For our work, it is plausible that the three conditions (Planktonic, Biofilm, CPB_Biofilm) lead to similar numbers of bacteria in the feces while having a very different impact on mouse homeostasis. Indeed, the physiological states of the cells were very different between the three conditions and could explain the improvement in health status observed with the CPB_Biofilm condition, notably by conferring enhanced probiotic properties despite stress related to the GIT.

Another advantage of pectin beads is their ability to be degraded in the colon, which makes it possible to consider using them as a carrier to deliver probiotics there. At low pH, calcium-pectinate beads maintain their ionic bonds, keeping the bead matrix intact. At neutral pH (intestine and the colon), the release of the biofilm-like microcolonies occurred thanks to a combination of longer residence time and the activity of pectinolytic enzymes. Therefore, the release can be explained by the swelling of the beads followed by their degradation ^42–45^. Moreover, FISH analysis confirmed that pectin beads allow the release of bacteria as biofilm-like microcolonies in the colon. The release of bacteria also exerted a decrease in the production of pro-inflammatory cytokines, particularly IL-1β and IL-6 and an increase in the production of anti-inflammatory cytokine IL-10. These results suggest that the improvement in the overall health status observed in this DSS-mouse model is likely related to the release of biofilm-like microcolonies in the colon. Earlier reports described that biofilm lifestyle enhanced the anti-inflammatory activities of probiotic bacteria compared to planktonic cells^22,23^. The anti-inflammatory effects of biofilm-like microcolonies encased in CPB observed in this DSS-induced colitis mouse model could thus be explained by the transport and delivery of bacteria in biofilm form to their site of action.

This study described the development of a new pectin-based technology for the encapsulation of probiotic bacteria in the form of biofilm-like structures. This new formulation allows delivering bacteria displaying a biofilm phenotype that confers them stress resistance properties and enhanced positive host health benefits compared to planktonic cells and released biofilm cells. The transportation and delivery of biofilm-like microcolonies in the colon is an interesting way to increase the effectiveness of existing probiotics, and could also be applied to probiotics under development.

## Methods

### Bacterial strain, media, and culture conditions

*L. paracasei* ATCC334 were grown in Man-Rogosa-Sharpe medium (MRS; Conda) pH 5.8 at 28 °C for both the biofilm and planktonic cultures. Before carrying out all the experiments, frozen cells were subcultured twice in MRS medium pH 5.8 and incubated for 24 h at 28 °C. Planktonic cultures were prepared by adding the second subculture into fresh medium at 10^7^ CFU mL^−1^, and incubating at 28 °C until the stationary phase. Cells were harvested twice (10 min, 5000 × *g*) and resuspended in 150 mM NaCl solution. The sample was named Planktonic. Biofilm cultures were prepared in polystyrene microplates by adding the second subculture into fresh MRS at 10^7^ CFU mL^−1^. One milliliter per well was dispensed on a 24-well microtiter plate (Costar 3524, Corning Incorporated) that was then incubated at 28 °C for 24 h. Cells attached to the well walls were quantified as previously described (Aoudia et al., 2016). After incubation, the medium was removed from each well, and the plates were washed twice with 500 µL of 150 mM NaCl solution to remove loosely attached cells. One milliliter of 150 mM NaCl solution was added to each well before repeated pipetting to detach the biofilm. The sample was named Biofilm. For both conditions, the number of viable cells was quantified in log (CFU/g) by serial dilutions of the planktonic suspension and the recovered biofilm suspension before being spotted onto MRS agar pH 5.8.

### Preparation of calcium-pectinate beads (CPB)

Amidated low methoxyl pectin with an above 25% esterification degree (DE) and an above 21% amidation degree (DA) was used for this study (Unipectine™ OF305C, Cargill). As illustrated in Fig. 1, aqueous pectin solution (4% w/v) was added in distilled water and stirred by a magnetic stirrer for 3 h. Then, *L. paracasei* ATCC334 cells from the second subculture were dispersed (10^7^ CFU mL^−1^) in the pectin solution until a uniform suspension was obtained. The cell suspension was dispersed dropwise using a Buchi Encapsulator (B-390) into sterile 750 mM CaCl_2_ used as a hardening solution in order to produce small beads (frequency vibration = 700 Hz, liquid flow rate = 12 mL/min, the diameter of the nozzle used = 300 µm). The CPB_placebo beads were prepared without the presence of *L. paracasei* ATCC334. The formation of the beads is based on ionotropic gelation due to the interaction between carboxylate groups of galacturonic acid (the main component of the pectin) and calcium ions. After a gelation time of 20 min in CaCl_2_, the beads containing *L. paracasei* ATCC334 were washed three times with distilled water and immersed in MRS (pH 5.8) for incubation (from 0 to 48 hours) which allowed the formation of biofilm-like structures inside the beads (CPB_Biofilm). Lastly, the number of viable cells encapsulated in the beads was quantified in log (CFU/g) by adding 10 mL of sodium citrate (0.1 M) to 1 g of the beads and incubating for 10 min. This led to the sequestration of the calcium involved in the formation of the beads by the citrate ions to form insoluble calcium citrate, resulting in the disintegration of the 3D pectin network. The viable liberated cells were enumerated by the drop plate method after 48 h incubation at 37 °C in MRS agar pH 5.8. A viability control was carried out to check the absence of cell death during the incubation in 0.1 M sodium citrate.

### Confocal laser scanning microscopy (CLSM)

A few CPB_Biofilms previously incubated at 28 °C for 0, 15, 24, and 48 h were dispensed per well on a 96-well microtiter plate (Greiner BioOne with a micro-clear bottom 655090). Prior to image acquisition, CPB were fluorescently labeled with Syto 9 green-fluorescent nucleic acid stain (Molecular Probes, Invitrogen, France). After incubation for 10 min, the samples were placed on the motorized stage of a Leica SP8 AOBS confocal laser scanning microscope (CLSM) (LEICA Microsystems) at the MIMA2 microscopy platform (doi.org/10.15454/1.5572348210007727E12). All the CPB_Biofilms were scanned at 600 Hz with a 40 × 0.8 N.A. (Leica HCX Apo) water immersion objective lens with a 488 nm argon laser set at 25% intensity. Emitted fluorescence was recorded within the range of 500–600 nm to visualize Syto 9 green fluorescence. Three stacks of horizontal plane images (512 × 512 pixels corresponding to 119 × 119 μm) with a z-step of 1 μm were acquired for each biofilm at different areas in the well. Two independent experiments were performed for each strain. Image analyses of three-dimensional projections of biofilm structures were reconstructed with the Easy 3D function of the IMARIS 9.3 software (Bitplane, Switzerland). The number of beads analyzed was equal to 10 for each incubation time. CPB_placebos were used as control.

### Cryogenic-scanning electron microscopy (cryo-SEM)

Imaging was performed in an ultra-high-resolution SEM SU8230 (HITACH) equipped with a cryo-preparation system (cryo Quorum PP3010T) at the DimaCell platform (http://www.dimacell.fr/). Samples of CPB_Biofilms were vitrified in pasty nitrogen, metalized for 30 s at 5 mA and fractured in cryo Quorum PP3010T. CPB_placebos were used as control.

### Resistance to acidic and osmotic stress

For acidic stress, the CPB_Biofilms were incubated at 37 °C for 2 h in 35 mM NaCl solution at pH 1.5; while for osmotic stress, CPB_Biofilms were incubated at 37 °C for 24 h in MRS pH 5.8 containing 4.5 M NaCl. For both stresses, the pH was adjusted after the addition of the beads to 1.5 and 5.8 for acidic and osmotic stresses, respectively. Bacteria from both control conditions (Planktonic and Biofilm) were processed concurrently. It is important to note that the bacterial quantity was adjusted at 10^10^ CFU before applying stress for all the conditions. Thereafter, bacteria and beads were added in 10 mL of sodium citrate (0.1 M) to disintegrate the pectin matrix and allow cell release^46^. The viable log (CFU/g) was enumerated before and after stress by spotting onto MRS agar pH 5.8 and incubating at 37 °C for 48 h.

### Resistance to freeze-drying

CPB_Biofilms were freeze-dried (FreeZone 18 Liter, Labconco) as were bacteria from both control conditions (Planktonic and Biofilm). For these three conditions, the bacterial quantity was adjusted to 10^10^ CFU before freeze-drying. The bacteria and beads were first frozen at −80 °C and then dried by applying a pressure of 0.05 mbar and a condenser temperature of −55 °C, after which five temperature steps were applied (−40 °C, 0 °C, 10 °C, 20 °C and 30 °C). The freeze-dried bacteria and beads were placed into tubes containing 1.25 mL of MRS pH 5.8 and 3.75 mL of sodium citrate (0.1 M) and then placed at 37 °C for 2 h to disintegrate the pectin matrix and allow cell release. The viable log (CFU/g) was enumerated before and after freeze drying by spotting onto MRS agar pH 5.8 and incubating at 37 °C for 48 h.

### Resistance to GI media

A model simulating the gastrointestinal tract (GIT) in humans consisting of three digestive compartments divided into the mouth, stomach, and small intestine was applied. The simulated juices were prepared from previous works^47,48^. The chemical composition and pH of simulated salivary fluid (SSF), simulated gastric juice (SGJ), and simulated intestinal fluid (SIF) are presented in Supplementary Table 1. The mouth step was defined as 5 min at 37 °C in the SSF adjusted to pH 7 and containing 1500 U mL^−1^ of α-amylase (A1031, Sigma). The gastric step was then conducted at 37 °C for 2 h in the SGJ containing 3 g L^−1^ of porcine pepsin (P7000, Sigma) and adjusted to pH 2. Finally, the duodenum step was carried out over 2 h in the SIF at pH 7 and included the addition of pancreatic enzymes at 1 g L^−1^ (P7545, Sigma) and bile fluid containing 45 g L^−1^ of bile salts (B8381, Sigma). After each step of the simulated digestion process, samples of each condition (Planktonic, Biofilm and CPB_Biofilm) were collected and the viable log (CFU/g) was enumerated by spotting onto MRS agar pH 5.8 and incubating at 37 °C for 48 h.

### Animals and experimental design

Forty-eight 8 week old male C57BL/6J mice were purchased from Charles River (France). Mice were housed in a temperature and humidity-controlled facility with a 12-h/12-h light/dark cycle. They were fed a standard chow diet and bred according to the local guidelines. All the experiments were approved by the local Animal Experimental Ethics committee, University of Burgundy, Dijon, France. Mice were randomly divided into six different groups (*n* = 8 mice in each group): control, DSS-treated, CPB_placebo DSS-treated and *L. paracasei* DSS-treated groups (Planktonic, Biofilm and CPB_Biofilm) (Supplementary Fig. 3). *L. paracasei*-treated mice were given 10^9^ bacteria in 0.1 mL 150 mM NaCl solution. This bacterial suspension was given daily by force-feeding for 15 days. Acute colitis was induced by the addition of 2% (w/v) dextran sodium sulfate (DSS) (molecular weight 36,000–50,000 Da; MP Biomedicals) dissolved in drinking water, which was fed ad libitum for 7 days between days 5 and 12^25^. Control C57BL/6 mice received the same drinking water without DSS. After 15 days, the animals were sacrificed.

### *L. paracasei* enumeration in mouse feces

Fresh feces were collected from the mice every other day just prior to administration of *L. paracasei* ATCC334. The feces were immediately weighed, diluted with 150 mM NaCl solution in a ratio of 15 mg stool to 0.45 ml 150 mM NaCl solution. The mixtures were then vortexed at a maximum speed for 2 min using three 1 mm-glass beads before being centrifuged at 800 × *g* for 3 min. The total number of colony forming units (CFU) was determined by plating serial dilutions of the supernatant on M-RTLV agar^49^ and incubating at 37 °C for 48 h. The results are expressed in CFU per gram wet weight.

### Fluorescent in situ hybridization (FISH)

Colon tissues were immediately fixed in Carnoy’s solution (6:3:1 (v/v) ethanol: acetic acid: chloroform) for at least 4 h at 4 °C to preserve the mucus layer^50^. These samples were then placed in 100% ethanol and embedded in paraffin at the CellimaP platform (http://www.cellimap.fr/). Prior to FISH analysis, sections of 3-µm thickness were deparaffinized by immersing the slides in xylene for 10 min and then in a mixture ethanol/xylene (1:1) for 3 min, as previously described with some modifications^51^. A series of hydration steps was performed in decreasing concentrations of ethanol (100, 95, 70, and 50% for 3 min each) and then the slides were washed with water. Subsequently, a lysozyme treatment was used (10 g L^−1^) in Tris–HCl (20 mmol L^−1^) at pH 6.5 for 10 min at 37 °C to enhance the permeabilization of the thick cell wall of lactobacilli, followed by a short wash with water. The hybridization of probes was carried out overnight at 48 °C with hybridization buffer containing lysozyme (10 g L^−1^), Tris–HCl (20 mmol L^−1^), NaCl (3.4 mmol L^−1^) at pH 8. All the fluorescently labeled probes were obtained from Eurogentec (Eurogentec S.A.) (Supplementary Table 2). After hybridization, the slides were washed with Tris–HCl (20 mmol.L^−1^), NaCl (0.9 mol.L-1) at pH 8 containing DAPI (dilution 5/1000) for 10 min at 50 °C. The slides were observed with a Leica SP8 AOBS Confocal laser scanning microscope (LEICA Microsystems) at the DimaCell platform (http://www.dimacell.fr/). After the acquisition, five images were analyzed for each condition using imageJ v1.53c software.

### Assessment of colitis

The severity of colitis was assessed from day 5 to 15 on the basis of clinical parameters (weight loss, stool consistency and presence of blood in feces) and presented as mouse weight loss (%) and a disease activity index (DAI) score with a maximum of 12, as previously described^52^. The following parameters were used for calculation: (a) weight loss (0 point = none, 1 point = 1–5% weight loss, 2 points = 5–10% weight loss, 3 points = 10–15% weight loss and 4 points = more than 15% weight loss), (b) stool consistency/diarrhea (0 points = normal, 2 points = loose stools, 4 points = watery diarrhea), (c) presence of blood in feces (0 = negative; 2 = bleeding; 4 = significant bleeding). The DAI was calculated as the total of these scores: the sum of weight loss, diarrhea and bleeding, resulting in a total DAI score ranging from 0 (unaffected) to 12 (severe colitis).

### Histology

Colon tissues were immediately fixed in Carnoy’s solution (6:3:1 (v/v) ethanol: acetic acid: chloroform) for at least 4 h at 4 °C, then placed in 100% ethanol and embedded in paraffin at the CellimaP platform (http://www.cellimap.fr/). The paraffin block was sliced at a thickness of 3 μm, stained with hematoxylin-eosin (H&E) and observed with an Olympus BX51 microscope.

### Cytokine expression

Colon tissue was transferred to a 2 mL reinforced tube (Precellys Lysing Kit) containing 1.4 mm ceramic beads (zirconium oxide, Precellys Lysing Kit) and 1 ml TRIzol Reagent (Invitrogen). Tissue was disrupted in a Precellys homogenizer (Bertin instruments) for 2 × 30 s at 6500 rpm. Nucleic acids were extracted in 0.2 volume of chloroform (Sigma) and purified by precipitation in 0.5 volume of isopropanol (Sigma). RNA pellets were dried and resuspended in 40 µL of RNAse-free water. Nucleic acid concentrations were determined by measuring absorbance at 260 nm using a NanoDrop spectrophotometer (ThermoScientific). One microgram of total RNA was treated for reverse transcription with the High Capacity cDNA Reverse Transcription kit (Applied Biosystems) according to the manufacturer’s instructions. Quantitative PCR was performed with iTaq Universal SYBER Green Supermix (Biorad) and the primer sets listed in Supplementary Table 3. The thermal cycling conditions comprised 30 s at 95 °C and then 40 cycles of 95 °C for 10 s and 60 °C for 30 s, followed by a standard melting curve analysis. Gene expression values were calculated based on the ΔΔCt method, ΔCt values were calculated using the Ct values from the amplification of a reference gene (GAPDH, housekeeping gene). Quantitative real-time PCR was performed on the CFX96 PCR system (Biorad).

### Cytokine assay

Blood samples were collected by intra-orbital puncture using a heparin-coated tube to obtain the plasma. Samples were centrifuged directly and plasma was isolated and stored at −80 °C until analysis. A multiplex sandwich immunoassay from the Bioplex protein array system (MO Th17 PANEL A GP1, 6-PLX, Bio-Rad), which contains fluorescence-labeled microspheres conjugated with monoclonal antibodies specific to 6 target cytokines, was used. Plasma samples were thawed and run in duplicates. Antibody-coupled beads were incubated with the plasma sample (antigen) after which they were incubated with biotinylated secondary (detection) antibody before finally being incubated with streptavidin-phycoerythrin. A range of standards were used to quantify cytokine concentrations. Bound molecules were then read by the Bio-Plex array reader which uses Luminex fluorescent-bead-based technology (Bioplex 200, Biorad) at the Cytometrie plateform (http://www.cytometrie-dijon.fr/). The analytes measured included IL-1β, IL-6, IL-10, IL-17, IFN-γ, TNF-α.

### Statistical analysis

Data are presented as mean ± standard deviation. Comparisons between groups were performed with the Kruskal–Wallis test using GraphPad Prism v8.2.4 software. A *p* value < 0.05 was considered significant.

### Reporting summary

Further information on experimental design is available in the Nature Research Reporting Summary linked to this paper.

## Supplementary information

Supplementary Information

Reporting Summary Checklist

## Data Availability

Data supporting the findings of this study are available within the paper and its Supplementary Information files. All other data are available from the corresponding author on request.
