# Supplementary material for: Intestinal release of biofilm-like microcolonies encased in calcium-pectinate beads increases probiotic properties of Lacticaseibacillus paracasei
Source: NPJ Biofilms Microbiomes. 2020 Oct 28;6:44. doi: 10.1038/s41522-020-00159-3 (PMC7595111; doi:10.1038/s41522-020-00159-3)
Supplement: Supplementary file 1 — Supplementary Information [file 41522_2020_159_MOESM1_ESM.pdf]

**Supplementary Materials for**

# Intestinal release of biofilm-like microcolonies encased in calcium pectinate beads increases probiotic properties of *Lacticaseibacillus paracasei*

Arnaud Heumann[a], Ali Assifaoui[a], David Da Silva Barreira[a], Charles Thomas[b,c], Romain Briandet[d], Julie Laurent[a], Laurent Beney[a], Pierre Lapaquette[a], Jean Guzzo[a] and Aurélie Rieu[a]

[a] *Université de Bourgogne Franche-Comté (UBFC), AgroSup Dijon, UMR PAM A 02.102, F-21000 Dijon, France.*

[b] *Université de Bourgogne Franche-Comté (UBFC), LNC UMR 1231, F-21000 Dijon, France; INSERM, LNC UMR 1231, F-21000 Dijon, France.*

[c] *Université de Bourgogne Franche-Comté (UBFC), LipSTIC LabEx, F-21000 Dijon, France.*

[d] *Université Paris-Saclay, INRAE, AgroParisTech, Micalis Institute, 78350, Jouy-en-Josas, France.*

Correspondence: Aurélie Rieu (aurelie.rieu@u-bourgogne.fr) and Ali Assifaoui (ali.assifaoui@u-bourgogne.fr)

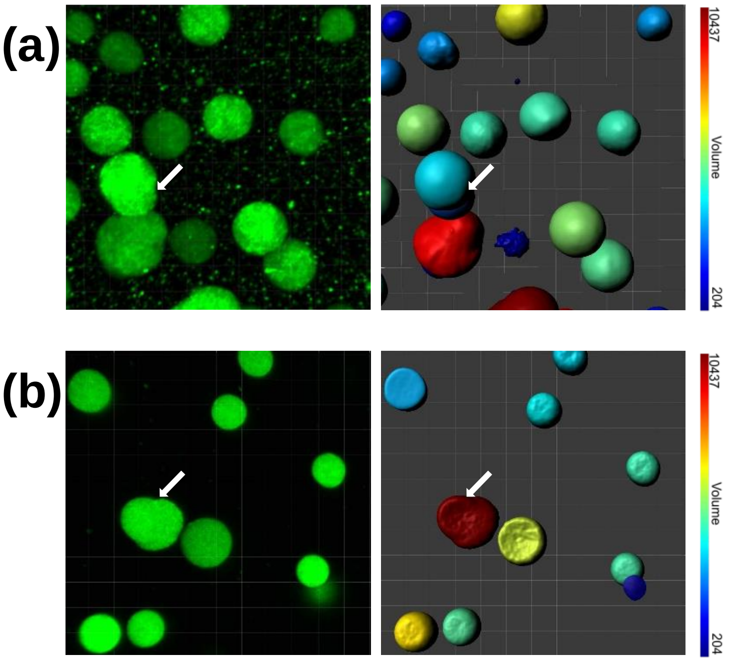


**Supplementary Figure 1.** Images of CPB_Biofilms obtained by laser scanning confocal microscopy (CLSM) at 24 h (**a**) and 48 h (**b**) in MRS media. In order to visualize size diversity and microcolony coalescence, fluorescence images obtained were transformed into isosurface representations with a coded color associated to the microcolony volume.

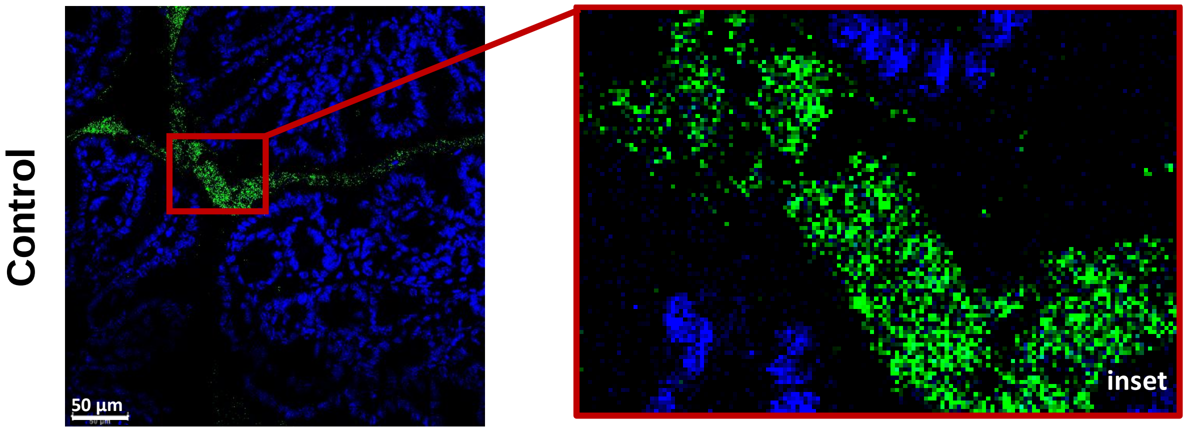


**Supplementary Figure 2.** *In situ* hybridization with fluorescence-labeled oligonucleotide probes. Colon sections of mice not fed by *L. paracasei* ATCC334 were hybridized with a *Lacticaseibacillus*-specific oligonucleotide probe (red) and a bacterium-specific oligonucleotide probe (green). *L. paracasei* ATCC334 cells appear red while all other bacteria appear green. Host nuclei were labeled with DAPI (blue).

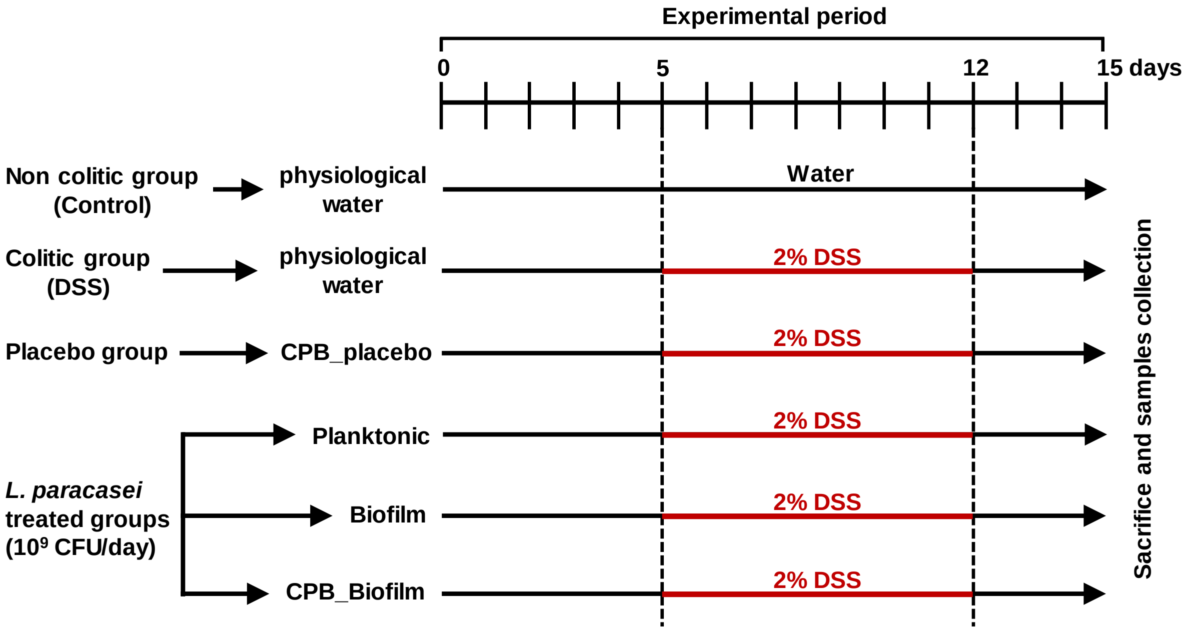


**Supplementary Figure 3.** *L. paracasei* ATCC334 treatment protocol for the relief of DSS-induced experimental colitis in mice. Daily, the *L. paracasei*-treated groups received intragastrically 100 µL of physiological water containing bacteria either in planktonic form, or in biofilm form or encapsulated as biofilm-like cultures in calcium-pectinate beads (CPB_Biofilm) over the 15-day experimental period. The placebo group received 100 µL of physiological water containing calcium-pectinate beads prepared without the presence of bacteria (CPB_placebo) and the colitic group received 100 µL of physiological water. Acute colitis was induced by the administration of 2% DSS in drinking water for 7 days (days 5-12). The non-colitic control group was not challenged with DSS and received 100 µL of physiological water.

**Supplementary Table 1.** Chemical composition and pH used for the different compartments of the *in vitro* simulated gastrointestinal digestion.

| | $mmol.L^{-1}$ | Simulated salivary fluid (SSF) pH 7 | Simulated gastric juice (SGJ) pH 2 | Simulated intestinal fluid (SIF) pH 7 |
|---|---|---|---|---|
| Chemical composition | KCl | 15.1 | 6.9 | 6.8 |
| | $KH_2PO_4$ | 3.7 | 0.9 | 0.8 |
| | $NaHCO_3$ | 13.6 | 25 | 85 |
| | NaCl | - | 47.2 | 38.4 |
| | $MgCl_2(H_2O)_6$ | 0.15 | 0.1 | 0.33 |
| | $(NH_4)_2CO_3$ | 0.06 | 0.5 | - |
| pH | HCl | 1.1 | 15.6 | 8.4 |

**Supplementary Table 2.** Fluorescent probes used for fluorescence in situ hybridization analysis.

| Probe | Specificity | Label | Sequence | References |
|---|---|---|---|---|
| Eub338 | Bacteria | Alexa fluor A488 | 5’-GCTGCCTCCCGTAGGAGC-3’ | 1 |
| Lpara | Lactobacilli | Alexa fluor A700 | 5’-GTTCCATGTTGAATCTCGG-3’ | 2 |

**Supplementary Table 3.** Primer sequences used for the amplification of cytokine genes and the housekeeping gene in the quantitative PCR.

| Gene | Forward Primer | Reverse Primer |
|---|---|---|
| TNF-α | 5’-GGTGCCTATGTCTCAGCCTC-3’ | 5’-GCTCCTCCACTTGGTGGTTT-3’ |
| Il-1β | 5’-GCCACCTTTTGACAGTGAT-3’ | 5’-GACAGCCCAGGTCAAAGGTT-3’ |
| IL-10 | 5’-TAACTGCACCCACTTCCCAG-3’ | 5’-AAGGCTTGGCAACCCAAGTA-3’ |
| GAPDH | 5’-ACCCAGAAGACTGTGGATGG-3’ | 5’-ACACATTGGGGGTAGGAACA-3’ |

## Supplementary References


1 Amann, R. I., Krumholz, L. & Stahl, D. A. Fluorescent-oligonucleotide probing of whole cells for determinative, phylogenetic, and environmental studies in microbiology. *J. Bacteriol.* **172**, 762-770 (1990).

2 Blasco, L., Ferrer, S. & Pardo, I. Development of specific fluorescent oligonucleotide probes for *in situ* identification of wine lactic acid bacteria. *FEMS Microbiol. Lett.* **225**, 115-123 (2003).
